# Discounting of delayed rewards: Missing data imputation for the 21- and 27-item monetary choice questionnaires

**DOI:** 10.1371/journal.pone.0292258

**Published:** 2023-10-16

**Authors:** Yu-Hua Yeh, Allison N. Tegge, Roberta Freitas-Lemos, Joel Myerson, Leonard Green, Warren K. Bickel

**Affiliations:** 1 Psychology Department, Illinois College, Jacksonville, IL, United States of America; 2 Fralin Biomedical Research Institute at VTC, Roanoke, VA, United States of America; 3 Department of Statistics, Virginia Tech, Blacksburg, VA, United States of America; 4 Department of Psychological and Brain Sciences, Washington University in St. Louis, St. Louis, MO, United States of America; Chinese Academy of Medical Sciences and Peking Union Medical College, CHINA

## Abstract

The Monetary Choice Questionnaire (MCQ) is a widely used behavioral task that measures the rate of delay discounting (i.e., *k*), the degree to which a delayed reward loses its present value as a function of the time to its receipt. Both 21- and 27-item MCQs have been extensively validated and proven valuable in research. Different methods have been developed to streamline MCQ scoring. However, existing scoring methods have yet to tackle the issue of missing responses or provide clear guidance on imputing such data. Due to this lack of knowledge, the present study developed and compared three imputation approaches that leverage the MCQ’s structure and prioritize ease of implementation. Additionally, their performance was compared with mode imputation. A Monte Carlo simulation was conducted to evaluate the performance of these approaches in handling various missing responses in each observation across two datasets from prior studies that employed the 21- and 27-item MCQs. One of the three approaches consistently outperformed mode imputation across all performance measures. This approach involves imputing missing values using congruent non-missing responses to the items corresponding to the same *k* value or introducing random responses when congruent answers are unavailable. This investigation unveils a straightforward method for imputing missing data in the MCQ while ensuring unbiased estimates. Along with the investigation, an R tool was developed for researchers to implement this strategy while streamlining the MCQ scoring process.

## Introduction

Delay discounting, the decrease of the present value of a delayed reward with the increase in the time to its receipt, captures important human decision-making processes [1]. An individual’s delay discounting rate, measured by the parameter *k* in a hyperbolic discounting model proposed by Mazur [2], is associated with various maladaptive behaviors, including substance addiction, gambling, and obesity [3–5]. Emerging evidence suggests delay discounting is not simply an indicator of poor cognitive functioning or a personality trait of impulsivity [6]. Noticeably, delay discounting has been proposed as a candidate behavioral marker of addiction and obesity and a transdiagnostic process in psychiatric disorders, highlighting its unique role in clinical research [7–9].

The Monetary Choice Questionnaire (MCQ) is one of the commonly used behavioral tasks that measure individuals’ rates of delay discounting [5, 10–13] is currently included in the PhenX Toolkit (https://www.phenxtoolkit.org), a catalog providing recommendations on data collection protocols for biomedical research. Two validated versions, the 21- and 27-item MCQs, are available [14, 15]. Both versions comprise a series of binary choice questions corresponding to different rates of delay discounting (i.e., *k*) based on Mazur’s hyperbolic discounting model, *V* = *A* / (1 + *kD*), where *V* is the present, discounted value, *A* is the amount of the delayed reward, *D* is the delay to its receipt, and *k* is a parameter governing the rate at which value is discounted with delay [2]. For example, a choice question “$34 tonight or $35 in 43 days” in the 21-item MCQ corresponds to a *k* value of 0.0007; a choice question “$31 today or $85 in 7 days” in the 27-item MCQ corresponds to a *k* value of .25. By analyzing individuals’ choice patterns on the questionnaire, the individual *k* values can be estimated.

The utility of the MCQ is evident from the amount of research in various fields, such as addiction, education, and health [16–18]. Moreover, the MCQ is a pragmatic and efficient assessment that measures the same construct as lengthier adjusting-based behavioral tasks (e.g., titrating the amount of the immediate reward to approximate the subjective value of a delayed reward), making it particularly useful in clinical applications [10, 19]. Additionally, the MCQ is low-cost and easy to administer [5, 11, 12] and can be completed either by paper and pencil or through computerized programs. The wide application of the MCQ merits further research to enhance the rigor of its measurement, which motivates the current investigation. Like many other behavioral tasks, researchers frequently need to deal with missing data in the MCQ when forcing a response is not possible or required. Even when a response can be forced, a participant may choose to discontinue for various reasons (e.g., indifferent between the choice options) and leave an incomplete observation. Significant challenges arise in the presence of missing data, as any number of missing responses interferes with the MCQ scoring. While the methodologies for handling missing data have evolved and improved significantly in recent decades, their utilization is not completely automated, which can pose a hurdle to their implementation [20]. Finding a balance between the trade-off of complexity and convenience in missing data imputation relies on researchers’ discretion, especially due to the lack of a consensus on the best practice for addressing missing responses in the MCQ. Commonly, researchers choose to exclude such data from the analysis, although this approach inevitably reduces statistical power (e.g., [21–23]). In order to address this challenge, the present study aims to provide guidance on handling missing values in the MCQ by evaluating different imputation approaches that emphasize ease of implementation.

The complexity involved in scoring the MCQ should be recognized. Different tools have been developed to automate the scoring process and reduce the risk of human error. For example, Kaplan et al. [24] developed freely available Excel-based scoring spreadsheets for both the 21- and 27-item MCQs. Their tool provides comprehensive information about the individual scores, including the *k* value, its log and natural log transformation, and the summary statistics. However, their tool is limited to scoring up to 1000 responses at a time. Gray et al. [11] developed the syntax to score both the 21- and 27-item MCQs in SPSS and R. Essentially, their tool conducts lookup operations using premade tables for any given choice patterns. Myerson et al. [25] proposed scoring the MCQ by calculating the proportion of the items in which a larger delayed reward is selected. Their method significantly simplifies the scoring process, and the proportional measure is highly correlated with the *k* value derived from the MCQ. Although convenient scoring methods are now available, none address missing responses. Some recommend data deletion if any or more than one response is missing for scoring [11, 24].

The structure of the MCQ may be capitalized on to conveniently impute missing responses. Specifically, the items in the MCQ can be grouped into three amount sets (i.e., small, medium, and large), which were intended to investigate the effect of the amount on the rate of delay discounting. These small, medium and large items may be thought of as three alternative forms of the same questionnaire. Thus, an individual *k* can be estimated with one of the three amount sets. Alternatively, a missing response to an item may be imputed with the non-missing responses to the items in the other amount sets. With this general concept, we developed three novel approaches to imputing missing data in the MCQ (see Methodology section).

The objectives of the current study were to provide empirical guidance on and a practical tool for imputing missing data in the MCQ. To achieve this, we compared three different imputation approaches capitalizing on the amount set structure with mode imputation, a simple method for handling missing values in binary variables. Our investigation focused on whether these approaches prioritizing ease of implementation would yield unbiased estimates and, if so, which would produce the most accurate estimates. Specifically, a Monte Carlo simulation was conducted to evaluate the performance of each imputation approach in handling different numbers of missing responses. The findings from this investigation offer valuable insights for researchers seeking an effective imputation approach for their MCQ data analysis and contribute to enhancing the rigor of delay discounting measurement.

## Methods

### Monetary choice questionnaire

Both 21- and 27-item MCQs comprise a series of dichotomous choices between smaller-immediate and larger-delayed hypothetical monetary rewards [14, 15]. By design, each item in the MCQ corresponds to a *k* value with which the two options (i.e., the smaller-immediate and the larger-delayed rewards) are subjectively equal according to the simple hyperbolic discounting model [2]. In addition, the items are divided into three sets based on whether the delayed amounts are small, medium, or large, and similar *k* values are used across the three sets. The items are ordered based on their associated *k* values from the smallest to the largest to score the MCQ. Specifically, when a choice pattern involves only one switching point at which a preference for a smaller-immediate reward changes to that for a larger-delayed reward, the individual *k* estimate can be inferred to be between the *k* values of the items where the switch occurs.

When a choice pattern involves more than one switching point, a consistency score that considers all questionnaire responses must be calculated for each item. The calculation consists in counting the instances of choosing the smaller-immediate amount before the given *k* value and the instances of choosing the larger-delayed amount at and following the given *k* value. The items with the greatest consistency scores will be used to infer the individual *k* estimate. Noticeably, because the items in the MCQ can be grouped into three sets by amount, a single choice pattern to the questionnaire can generate at least four different *k* estimates (i.e., one *k* value derived for all the items, and *k* values for items for the small, medium, and large delayed amounts). An additional fifth *k* estimate can also be derived by calculating the geometric mean of the resulting *k* values for the small, medium, and large amounts (i.e., a composite value). Following the terms used in Kaplan et al. [24], we refer to these five *k* values as overall, small, medium, large, and composite *k*s throughout this article.

We followed the Excel-based tool developed by Kaplan et al. [24] to develop an MCQ scorer in R. We also added new functions to allow our tool to impute missing data. These new functions are based on the imputation approaches described below. The R scoring tool, detailing every step in the procedure, is freely available at https://osf.io/p29uk/, and instruction on how to use it is provided in the supplementary document of this article (see S1 Appendix).

### Imputation approaches

#### Approach 1 –mode imputation

This imputation approach substitutes each missing value with the mode of the responses to the corresponding item. For example, if the majority of responses to “$31 today or $85 in 7 days” in a given sample is “$85 in 7 days”, this response is used to replace any missing responses for this item.

#### Approach 2 –group geometric mean (GGM)

The standard scoring procedure calculates the composite *k* only when all small, medium and large *k*s are available. This imputation approach relaxes this prerequisite and calculates the composite *k* when at least one of the three amount set *k*s is fully available. For example, if the small *k* cannot be derived due to missing responses, the composite *k* will be calculated with the medium and large *k*s; if both small and medium *k*s cannot be derived due to missing responses, the composite *k* will be equal to the large *k*. In sum, this imputation approach permits the estimation of an individual composite *k* when the missing responses do not appear in all three amount sets.

#### Approach 3 –item nearest neighbor (INN) without random

This imputation approach replaces the missing value with the congruent non-missing responses to the items corresponding to the same *k* value. For example, in the 21-item MCQ, if the response to “$34 tonight or $35 in 43 days” (small amount) is missing, then the responses to “$53 tonight or $55 in 55 days” (medium amount) and “$83 tonight or $85 in 35 days” (large amount) will be referenced because all three items correspond to the same *k* value (i.e., 0.0007). Suppose the responses to the medium and large amount items are congruent (i.e., choosing immediate or delayed rewards for both items). In that case, the same choice will be assumed for the small amount item. However, if the responses to the medium and large amount items are incongruent, the response to the small amount item will be left missing. Notice that in the case where only one item could be referenced (e.g., the responses to both the medium and large amount items are missing, but the response to the small amount item is non-missing), the single non-missing data will replace the missing responses to the other items corresponding to the same *k* value.

#### Approach 4 –item nearest neighbor (INN) with random

This imputation approach is identical to approach 3, except that when a missing response cannot be resolved, this datum will be randomly replaced with 0 or 1, corresponding to choosing immediate or delayed rewards, respectively. As such, no data will be missing when this imputation approach is implemented.

### Approach evaluation

#### Datasets

To evaluate the performance of each imputation approach, two datasets from previously published studies were utilized [26, 27]. The first and the second datasets comprise 900 and 512 complete observations (i.e., no missing data) for the 21- and 27-item MCQs, respectively. In both studies, a choice questionnaire with a similar structure to the MCQ was developed to measure probability discounting, decreasing the subjective value of a probabilistic reward as the likelihood of its occurrence decreases. The permissions to use the datasets were obtained from contacting the corresponding authors. Both datasets were de-identified, and only the information relevant to the analysis in this study was included.

#### Monte Carlo simulation

Monte Carlo simulation is a *what-if* analysis that relies on repeated random sampling to quantify the uncertainty associated with different data conditions [28]. In this study, we examined the performance of each imputation approach in handling different numbers of missing responses (*r*s) to the MCQ, ranging from 1 to 5 for the 21-item version and from 1 to 7 for the 27-item version (approximately 25% of the items in each version), with 1000 iterations. In each iteration, a series of performance measures were computed for the 12 conditions (5 conditions for the 21-item version and 7 conditions for the 27-item version). Specifically, under each condition, a fixed number of responses was randomly chosen and removed for each observation, which resulted in a dataset with missing data. Each imputation approach was then applied to this dataset to obtain individual composite *k* values. The mean difference between the composite *k*s derived from the datasets with and without missing data was calculated as a performance measure at the group level. The root-mean-square deviation (RMSD), the square root of the mean of the squares of the differences between the actual and imputed composite *k*s, was calculated as a measure of performance at the individual level. Furthermore, the correlation between the true and imputed composite *k*s was computed. Unlike mode imputation (approach 1) and INN with random (approach 4), the ability of GGM (approach 2) and INN without random (approach 3) to impute a dataset depends on the pattern of missing responses across the amount sets. Thus, the proportion of the observations that these approaches for each dataset could not handle was also calculated. Finally, to evaluate the influence of the imputation approaches on changing the delay discounting research results, the correlation between the natural logarithmic composite *k* (to approximate a normal distribution) and the probability discounting measure was calculated and pitted against the true value from the complete dataset.

## Results

**Fig 1:**
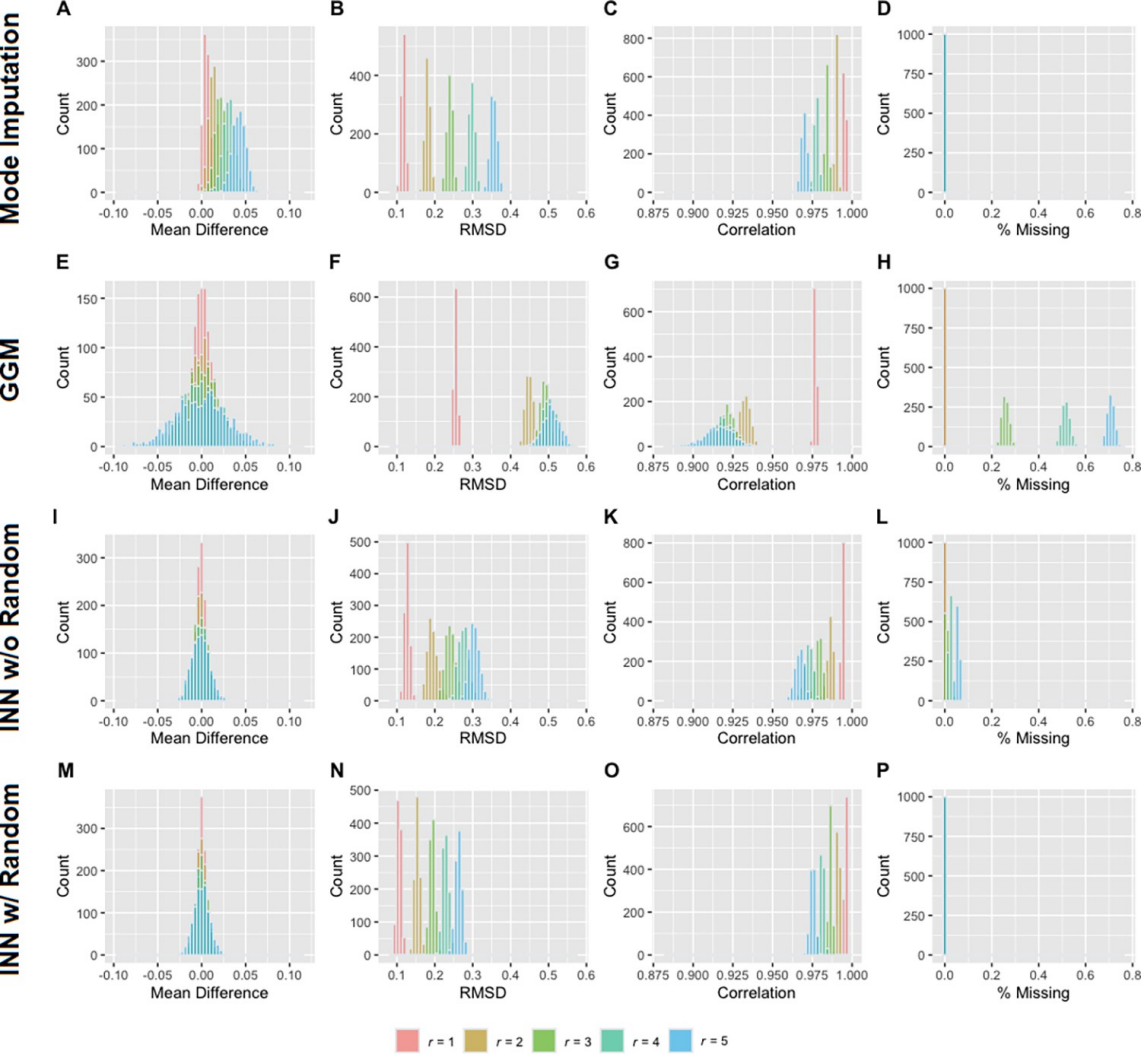
Performance of each imputation approach for the 21-item MCQ. Panels A-D, E-H, I-L, and M-P are the results from mode imputation (approach 1), group geometric mean (GGM; approach 2), item nearest neighbor without random (INN w/o Random; approach 3) and item nearest neighbor with random (INN w/ Random; approach 4), respectively. RMSD = root-mean-square deviation; *r* = number of missing responses in each observation.

**Fig 2:**
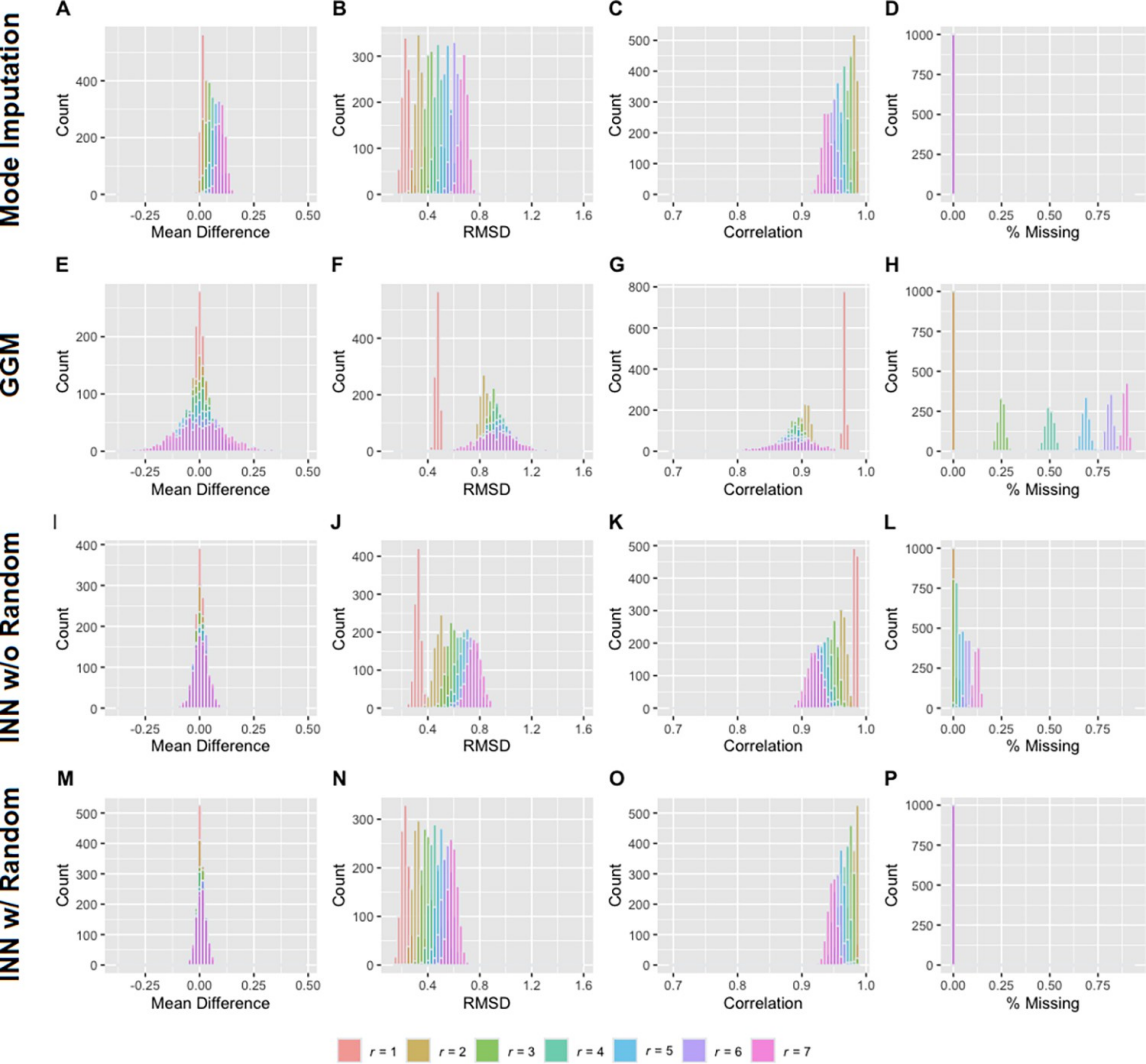
Performance of each imputation approach for the 27-item MCQ. Panels A-D, E-H, I-L, and M-P are the results from mode imputation (approach 1), group geometric mean (GGM; approach 2), item nearest neighbor without random (INN w/o Random; approach 3) and item nearest neighbor with random (INN w/ Random; approach 4), respectively. RMSD = root-mean-square deviation; *r* = number of missing responses in each observation.

**Fig 3:**
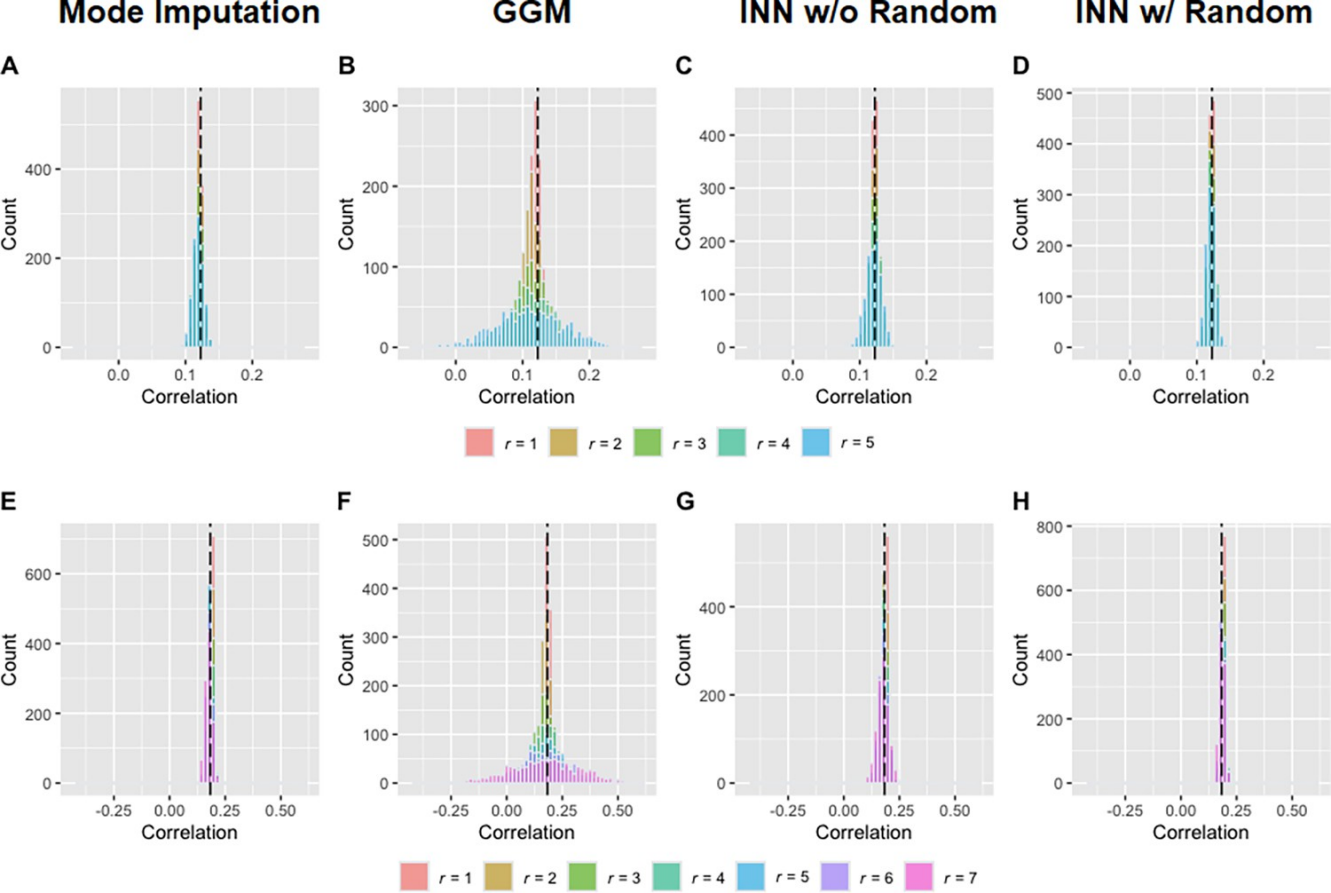
The distributions of the correlations between the imputed estimates and the probability discounting for the (A-D) 21-item and (E-H) 27-item MCQs. Panels A and E, B and F, C and G, and D and H are the results from mode imputation (approach 1), group geometric mean (GGM; approach 2), item nearest neighbor without random (INN w/o Random; approach 3) and item nearest neighbor with random (INN w/ random; approach 4), respectively. The black solid vertical lines indicate the observed correlations calculated from the original datasets. r = number of missing responses in each observation.

Figs 1 and 2 depict the results of the simulations for the 21- and 27-item MCQs, respectively. Meanwhile, Tables 1 and 2 provide a summary of mean and standard deviation of each distribution. As anticipated, with an increase in r, the deviations of the imputed composite k grew at both group and individual levels across all three approaches. As may be seen, the mean difference in mode imputation slightly increased with r. In contrast, the distributions of mean difference in the other imputation approaches were centered around 0, indicating unbiased estimation at the group level. Notably, across all performance measures (i.e., mean difference, RMSD, and correlation with the true score), INN with random (approach 4) consistently outperformed the other approaches and has the advantage of imputing all observations. Similarly, in the distributions of the correlation between the natural logarithmic composite *k* and probability discounting, INN with random and mode imputation exhibited comparable performance, yielding the least biased measure (see Fig 3).

**Table 1:** Average performance of imputation approaches across performance measures for the 21-item MCQ.

	r = 1	r = 2	r = 3	r = 4	r = 5	Overall
Mean difference from true scores						
Mode	0.005 (0.004)	0.013 (0.005)	0.021 (0.006)	0.031 (0.007)	0.042 (0.007)	0.023 (0.015)
GGM	-0.000 (0.008)	-0.000 (0.016)	0.001 (0.019)	-0.001 (0.024)	0.000 (0.031)	-0.000 (0.021)
INN without random	-0.001 (0.004)	-0.002 (0.006)	-0.001 (0.008)	-0.002 (0.009)	-0.001 (0.010)	-0.001 (0.008)
INN with random	0.000 (0.004)	-0.000 (0.005)	0.000 (0.006)	-0.000 (0.007)	-0.000 (0.009)	-0.000 (0.007)
Root-mean-square deviation from true scores						
Mode	0.117 (0.005)	0.181 (0.007)	0.240 (0.008)	0.297 (0.009)	0.355 (0.009)	0.238 (0.084)
GGM	0.255 (0.005)	0.449 (0.010)	0.489 (0.012)	0.504 (0.017)	0.508 (0.023)	0.441 (0.097)
INN without random	0.127 (0.006)	0.195 (0.014)	0.241 (0.014)	0.276 (0.014)	0.304 (0.013)	0.229 (0.064)
INN with random	0.106 (0.006)	0.154 (0.007)	0.193 (0.007)	0.229 (0.008)	0.263 (0.009)	0.189 (0.056)
Correlation with true scores						
Mode	0.995 (0.000)	0.990 (0.001)	0.984 (0.001)	0.978 (0.001)	0.970 (0.002)	0.983 (0.009)
GGM	0.977 (0.001)	0.933 (0.003)	0.922 (0.005)	0.917 (0.007)	0.916 (0.010)	0.933 (0.024)
INN without random	0.994 (0.001)	0.986 (0.002)	0.979 (0.002)	0.973 (0.003)	0.967 (0.003)	0.980 (0.010)
INN with random	0.996 (0.000)	0.991 (0.001)	0.986 (0.001)	0.981 (0.001)	0.975 (0.002)	0.986 (0.007)
Number of missing after imputation						
Mode	0 (0)	0 (0)	0 (0)	0 (0)	0 (0)	0 (0)
GGM	0 (0)	0 (0)	232 (13.5)	464 (15.8)	637 (13.7)	267 (253)
INN without random	0 (0)	0 (0)	5.35 (2.31)	20.0 (4.49)	49.2 (6.80)	14.9 (19.0)
INN with random	0 (0)	0 (0)	0 (0)	0 (0)	0 (0)	0 (0)
Difference in correlation with probability discounting						
Mode	-0.001 (0.003)	-0.002 (0.005)	-0.003 (0.006)	-0.004 (0.007)	-0.004 (0.008)	-0.003 (0.006)
GGM	-0.003 (0.007)	-0.009 (0.012)	-0.009 (0.023)	-0.009 (0.038)	-0.013 (0.054)	-0.009 (0.032)
INN without random	-0.000 (0.004)	0.000 (0.006)	-0.000 (0.007)	-0.001 (0.009)	-0.002 (0.012)	-0.001 (0.008)
INN with random	-0.000 (0.003)	-0.001 (0.004)	-0.001 (0.005)	-0.001 (0.006)	-0.002 (0.007)	-0.001 (0.006)

The number in the parenthesis is one standard deviation of the mean. *r* = number of missing responses in each observation; GGM = Group Geometric Mean; INN = Item Nearest Neighbor.

**Table 2:** Average performance of imputation approaches across performance measures for the 27-item MCQ.

	r = 1	r = 2	r = 3	r = 4	r = 5	r = 6	r = 7	Overall
Mean difference from true scores								
Mode	0.015 (0.010)	0.029 (0.014)	0.044 (0.015)	0.059 (0.017)	0.074 (0.018)	0.089 (0.018)	0.103 (0.019)	0.059 (0.034)
GGM	0.000 (0.022)	0.001 (0.037)	-0.000 (0.048)	-0.001 (0.057)	-0.004 (0.077)	-0.002 (0.098)	0.005 (0.125)	-0.000 (0.074)
INN without random	0.001 (0.014)	0.003 (0.021)	0.002 (0.026)	0.003 (0.028)	0.005 (0.031)	0.007 (0.032)	0.006 (0.035)	0.004 (0.028)
INN with random	0.002 (0.010)	0.004 (0.013)	0.005 (0.016)	0.006 (0.019)	0.008 (0.021)	0.009 (0.022)	0.008 (0.023)	0.006 (0.018)
Root-mean-square deviation from true scores								
Mode	0.232 (0.028)	0.333 (0.029)	0.411 (0.029)	0.481 (0.030)	0.548 (0.031)	0.610 (0.031)	0.676 (0.031)	0.470 (0.148)
GGM	0.475 (0.016)	0.834 (0.038)	0.907 (0.047)	0.934 (0.064)	0.940 (0.090)	0.943 (0.115)	0.929 (0.157)	0.852 (0.181)
INN without random	0.322 (0.024)	0.492 (0.042)	0.586 (0.046)	0.655 (0.048)	0.695 (0.050)	0.735 (0.051)	0.761 (0.053)	0.607 (0.151)
INN with random	0.222 (0.029)	0.317 (0.031)	0.387 (0.033)	0.448 (0.034)	0.502 (0.036)	0.548 (0.039)	0.595 (0.038)	0.431 (0.127)
Correlation with true scores								
Mode	0.992 (0.002)	0.983 (0.003)	0.975 (0.004)	0.966 (0.005)	0.957 (0.006)	0.948 (0.006)	0.938 (0.007)	0.965 (0.019)
GGM	0.966 (0.002)	0.906 (0.008)	0.891 (0.012)	0.886 (0.017)	0.884 (0.024)	0.882 (0.031)	0.882 (0.042)	0.900 (0.037)
INN without random	0.984 (0.002)	0.963 (0.006)	0.949 (0.008)	0.938 (0.009)	0.930 (0.010)	0.923 (0.011)	0.918 (0.011)	0.944 (0.024)
INN with random	0.992 (0.002)	0.984 (0.003)	0.977 (0.004)	0.969 (0.005)	0.962 (0.005)	0.954 (0.006)	0.947 (0.007)	0.969 (0.016)
Number of missing after imputation								
Mode	0 (0)	0 (0)	0 (0)	0 (0)	0 (0)	0 (0)	0 (0)	0 (0)
GGM	0 (0)	0 (0)	127 (9.49)	255 (11.1)	351 (10.4)	416 (8.91)	457 (6.80)	229 (176)
INN without random	0 (0)	0 (0)	3.05 (1.75)	9.67 (3.08)	20.9 (4.36)	37.8 (5.78)	61.9 (7.47)	19.0 (22.00)
INN with random	0 (0)	0 (0)	0 (0)	0 (0)	0 (0)	0 (0)	0 (0)	0 (0)
Difference in correlation with probability discounting								
Mode	0.009 (0.006)	0.006 (0.008)	0.004 (0.009)	0.002 (0.011)	-0.001 (0.012)	-0.005 (0.013)	-0.007 (0.015)	0.001 (0.012)
GGM	0.003 (0.012)	-0.008 (0.019)	-0.011 (0.040)	-0.012 (0.059)	-0.009 (0.084)	-0.011 (0.118)	-0.011 (0.157)	-0.008 (0.086)
INN without random	0.007 (0.008)	0.003 (0.012)	0.001 (0.015)	-0.002 (0.017)	-0.004 (0.019)	-0.006 (0.022)	-0.007 (0.026)	-0.001 (0.019)
INN with random	0.009 (0.005)	0.008 (0.007)	0.008 (0.009)	0.006 (0.010)	0.005 (0.010)	0.003 (0.011)	0.002 (0.012)	0.006 (0.010)

The number in the parenthesis is one standard deviation of the mean. r = number of missing responses in each observation; GGM = Group Geometric Mean; INN = Item Nearest Neighbor.

## Discussion

The aim of the current study was to evaluate three novel approaches designed to impute missing data in the 21- and 27-item MCQs and compare their performance with mode imputation. The Monte Carlo simulation with different numbers of missing responses in each observation revealed that INN with random (approach 4) consistently outperformed the other approaches, including mode imputation. This approach involves replacing the missing value with the congruent non-missing responses from items corresponding to the same *k* value. Any residual missing values are then replaced with random responses.

The fact that adding randomness to the data produced a more precise, unbiased estimate may appear puzzling. However, this unambiguous finding can be explained by the scoring procedure of the MCQ. Instead of deriving a score by summing responses like many other questionnaires, the scoring of the MCQ relies on the overall choice pattern that determines the consistency score of each item. Replacing a missing value with a random response has no effect on the individual *k* estimate unless that response would change the item with the highest consistency score. Noticeably, in the current study, INN without and with random (approaches 3 and 4) are mostly identical and would produce the same results if the former approach can fully impute a tested dataset. The advantage of INN with random observed in the performance measures is simply because this approach could impute all missing data across conditions. Such a finding indicates that adding randomness is more beneficial than removing observations to handle missing data in the MCQ, highlighting the importance of maximizing the sample size with imputation methods.

Unlike GGM (approach 2), which is designed to impute the individual *k* estimate, INN without and with random (approaches 3 and 4) are intended to impute the missing data at the item level, which makes them versatile. Specifically, alternative ways to score the MCQ without yielding a *k* value exist, such as calculating the proportion of the items in which a larger delayed reward is selected [25]. For researchers who opt to use alternative scoring approaches, both INN without and with random can still be used to impute the missing data. Although the current study did not evaluate the influence of these two imputation approaches on changing the alternative scores, similar outcomes can be assumed, considering the high correlations that have been observed between the *k* value and the alternative measures [25, 29]. The mode imputation is another convenient way to handle missing data in this regard. However, our simulation results recommend against its use because it may produce biased estimates.

Although not in the scope of this research, multilevel logistic modeling is a relevant approach to treating missing data and may be a preferred method to score the MCQ. The multilevel modeling approach makes inferences from all observed data and does not rely on imputation. When the assumption of missing completed at random (i.e., no systematic differences between the missing and the observed data) or missing at random (other observed variables can entirely explain the systematic differences between the missing and the observed data) is met, this approach is free from sample and estimate biases [30]. In addition, multilevel modeling eliminates any issues accompanied by the two-stage analytical approach in which individual discounting rates are determined and later used in subsequent analysis. However, multilevel logistic modeling significantly increases the overall complexity of the analysis, and the estimates may fail to converge [29, 31]. Moreover, this approach produces two separate coefficients, one for Amount and one for Delay, instead of a unified discount rate estimate (e.g., *k* value), which complicates the comparison with existing literature. Thus, some researchers may choose the conventional MCQ scoring tools over multilevel logistic modeling, and our imputation approaches are complementary to handling missing data in this scenario.

The present study possesses several limitations that warrant acknowledgment. Firstly, our evaluation of different imputation approaches is restricted to observations with up to 5 and 7 missing responses for the 21- and 27-item MCQs, respectively. Consequently, the precise imputation capacity of these approaches for observations with higher numbers of missing responses remains uncertain. Secondly, both datasets employed in this study consisted of online samples from general populations. As the MCQ holds clinical relevance, the generalizability of our findings to diverse populations necessitates further investigation. Thirdly, an imputation approach discussed by Gray et al. [11] that also capitalizes on the structure of the MCQ was not evaluated. That approach replaces the missing data with the response to the item with the closest *k* value *in the same amount set*. In other words, a response to the item corresponding to a different *k* value is used to replace the one that is missing. Given this approach will inevitably produce a biased estimate, we opted not to include it in the current investigation.

Finally, whether other generic imputation approaches such as logistic regression would perform better than INN with random remains unclear. Nonetheless, such investigation is out of the scope of the current study as our primary objective is to offer guidance on handling missing values in the MCQ while balancing complexity and convenience. In contrast to these more complex methods that often incorporate additional information, such as demographics or performance on other measures, the approaches examined in our study rely solely on MCQ data. This distinction underscores the simplicity of our chosen approaches, potentially promoting their adoption, enhancing the scientific integrity of measurement, and facilitating data reproducibility.

## Supporting information

S1 Appendix Instruction of the R tool for scoring the 21- and 27-item Monetary Choice Questionnaire (MCQ). (DOCX)

## References

[1] Green L, Myerson J. A discounting framework for choice with delayed and probabilistic rewards. Psychol Bull. 2004;130:769–92. doi: 10.1037/0033-2909.130.5.769 15367080 PMC1382186

[2] Mazur JE. An adjusting procedure for studying delayed reinforcement. In: Commons ML, Mazur JE, Nevin JA, editors. The effect of delay and of intervening events on reinforcement value. New Jersey: Lawrence Erlbaum Associates, Inc; 1987. p. 55–73.

[3] Amlung M, Petker T, Jackson J, Balodis I, MacKillop J. Steep discounting of delayed monetary and food rewards in obesity: a meta-analysis. Psychol Med. 2016;46:2423–34. doi: 10.1017/S0033291716000866 27299672

[4] Ioannidis K, Hook R, Wickham K, Grant JE, Chamberlain SR. Impulsivity in gambling disorder and problem gambling: a meta-analysis. Neuropsychopharmacology. 2019;44:1354–61. doi: 10.1038/s41386-019-0393-9 30986818 PMC6588525

[5] MacKillop J, Amlung MT, Few LR, Ray LA, Sweet LH, Munafò MR. Delayed reward discounting and addictive behavior: a meta-analysis. Psychopharmacology. 2011;216:305–21. doi: 10.1007/s00213-011-2229-0 21373791 PMC3201846

[6] Yeh Y-H, Myerson J, Green L. Delay discounting, cognitive ability, and personality: what matters? Psychon Bull Rev. 2021;28:686–94. doi: 10.3758/s13423-020-01777-w 33219456 PMC8068578

[7] Amlung M, Marsden E, Holshausen K, Morris V, Patel H, Vedelago L, et al. Delay discounting as a transdiagnostic process in psychiatric disorders: a meta-analysis. JAMA Psychiatry. 2019;76:1176–86. doi: 10.1001/jamapsychiatry.2019.2102 31461131 PMC6714026

[8] Bickel WK, Koffarnus MN, Moody L, Wilson AG. The behavioral- and neuro-economic process of temporal discounting: a candidate behavioral marker of addiction. Neuropharmacology. 2014;76 Pt B:518–27. doi: 10.1016/j.neuropharm.2013.06.013 23806805 PMC3858579

[9] Bickel WK, Freitas-Lemos R, Tomlinson DC, Craft WH, Keith DR, Athamneh LN, et al. Temporal discounting as a candidate behavioral Marker of Obesity. Neurosci Biobehav Rev. 2021;129:307–29. doi: 10.1016/j.neubiorev.2021.07.035 34358579

[10] Amlung M, Vedelago L, Acker J, Balodis I, MacKillop J. Steep delay discounting and addictive behavior: a meta-analysis of continuous associations. Addiction. 2017;112:51–62. doi: 10.1111/add.13535 27450931 PMC5148639

[11] Gray JC, Amlung MT, Palmer AA, MacKillop J. Syntax for calculation of discounting indices from the monetary choice questionnaire and probability discounting questionnaire. J Exp Anal Behav. 2016;106:156–63. doi: 10.1002/jeab.221 27644448 PMC5042866

[12] Matta A, Gonçalves FL, Bizarro L. Delay discounting: concepts and measures. Psychol Neurosci. 2012;5(2):135–46.

[13] Weinsztok S, Brassard S, Balodis I, Martin LE, Amlung M. Delay discounting in established and proposed behavioral addictions: a systematic review and meta-analysis. Front Behav Neurosci. 2021;15:786358. doi: 10.3389/fnbeh.2021.786358 34899207 PMC8661136

[14] Kirby KN, Maraković NN. Delay-discounting probabilistic rewards: rates decrease as amounts increase. Psychon Bull Rev. 1996;3:100–4. doi: 10.3758/BF03210748 24214810

[15] Kirby KN, Petry NM, Bickel WK. Heroin addicts have higher discount rates for delayed rewards than non-drug-using controls. J Exp Psychol Gen. 1999;128:78–87. doi: 10.1037//0096-3445.128.1.78 10100392

[16] Duckworth AL, Seligman MEP. Self-discipline outdoes IQ in predicting academic performance of adolescents. Psychol Sci. 2005;16:939–44. doi: 10.1111/j.1467-9280.2005.01641.x 16313657

[17] Kirby KN, Finch JC. The hierarchical structure of self-reported impulsivity. Pers Individ Dif. 2010;48:704–13. doi: 10.1016/j.paid.2010.01.019 20224803 PMC2834306

[18] Kirby KN, Petry NM. Heroin and cocaine abusers have higher discount rates for delayed rewards than alcoholics or non-drug-using controls. Addiction. 2004;99:461–71. doi: 10.1111/j.1360-0443.2003.00669.x 15049746

[19] Wan H, Myerson J, Green L. Individual differences in degree of discounting: do different procedures and measures assess the same construct? Behav Processes. 2023;208:104864. doi: 10.1016/j.beproc.2023.104864 37001683

[20] Enders CK. Missing data: an update on the state of the art. Psychol Methods. 2023. doi: 10.1037/met0000563 36931827

[21] Mishra S, Lalumière ML. Associations between delay discounting and risk-related behaviors, traits, attitudes, and outcomes. J Behav Decis Mak. 2017;30:769–81.

[22] Simmen-Janevska K, Forstmeier S, Krammer S, Maercker A. Does trauma impair self-control? Differences in delaying gratification between former indentured child laborers and nontraumatized controls. Violence Vict. 2015;30:1068–81. 26440574 10.1891/0886-6708.VV-D-13-00174

[23] Teti Mayer J, Nicolier M, Tio G, Mouchabac S, Haffen E, Bennabi D. Effects of high frequency repetitive transcranial magnetic stimulation (HF-rTMS) on delay discounting in major depressive disorder: an open-label uncontrolled pilot study. Brain Sci. 2019;9. doi: 10.3390/brainsci9090230 31514324 PMC6769715

[24] Kaplan BA, Amlung M, Reed DD, Jarmolowicz DP, McKerchar TL, Lemley SM. Automating scoring of delay discounting for the 21- and 27-item monetary choice questionnaires. The Behavior Analyst. 2016;39:293–304. doi: 10.1007/s40614-016-0070-9 31976983 PMC6701266

[25] Myerson J, Baumann AA, Green L. Discounting of delayed rewards: (a)theoretical interpretation of the Kirby questionnaire. Behav Processes. 2014;107:99–105. doi: 10.1016/j.beproc.2014.07.021 25139835 PMC4418201

[26] Jarmolowicz DP, Bickel WK, Carter AE, Franck CT, Mueller ET. Using crowdsourcing to examine relations between delay and probability discounting. Behav Processes. 2012;91:308–12. doi: 10.1016/j.beproc.2012.09.001 22982370 PMC4732266

[27] Yeh Y-H. Evaluating everyday behaviors with delayed and/or probabilistic consequences through a discounting framework [dissertation]. St. Louis (MO): Washington University in St. Louis; 2021.

[28] Mooney CZ. Monte Carlo simulation. New York: SAGE; 1997.

[29] Wileyto EP, Paul Wileyto E, Audrain-Mcgovern J, Epstein LH, Lerman C. Using logistic regression to estimate delay-discounting functions. Behav Res Methods. 2004;36:41–51. doi: 10.3758/bf03195548 15190698

[30] Gueorguieva R, Krystal JH. Move over ANOVA: progress in analyzing repeated-measures data and its reflection in papers published in the Archives of General Psychiatry. Arch Gen Psychiatry. 2004;61:310–17. doi: 10.1001/archpsyc.61.3.310 14993119

[31] Young ME. Discounting: a practical guide to multilevel analysis of indifference data. J Exp Anal Behav. 2017;108:97–112. doi: 10.1002/jeab.265 28699271

