# Supplementary material for: Discounting of delayed rewards: Missing data imputation for the 21- and 27-item monetary choice questionnaires
Source: PLoS One. 2023 Oct 16;18(10):e0292258. doi: 10.1371/journal.pone.0292258 (PMC10578570; doi:10.1371/journal.pone.0292258)
Supplement: S1 Appendix — (DOCX) [file pone.0292258.s001.docx]

# **S1 Appendix**

## **Instruction of the R tool for scoring the 21- and 27-item Monetary Choice Questionnaire (MCQ)**

The R scoring tool described in our research article is available for download via an online-curated digital repository at <https://osf.io/p29uk/>. This scoring tool has the “psych” package as the only prerequisite. Readers who are unable to perform basic operations such as loading an R package and executing an R script may want to familiarize themselves with the programming language by visiting the R official website (<https://www.r-project.org/>). We created our R scoring tool following Kaplan et al. (2016). All users are encouraged to read their work to learn how to interpret the variables included in the output.

Two R scripts may be found and downloaded from the online repository. The file “MCQ.scorer.R” includes all the scoring functions. Users may source this file and use the wrapper function, MCQ.scorer(), to score their dataset. This wrapper function takes three arguments: x, impute, and random. The first argument, x, is a data frame of the MCQ dataset to be scored. The first column of the data frame should be the index of the observations, and the following columns should be ordered in the same sequence as the questions being presented to the participants. In the data frame, the choice of the now options should be coded as 0, the choice of the delayed options should be coded as 1, and the missing responses should be coded as NA. The second argument, impute, has a default value, “none”, and can be replaced with “Mode”, “GGM”, or “INN”, which correspond to the imputation approaches Mode Imputation, Group Geometric Mean and Item Nearest Neighbor described in the research article, respectively. The third argument, random, has a default value, “FALSE”. When impute = “INN” and random = “FALSE”, the scoring tool will use Item Nearest Neighbor without random (approach 3) to impute the dataset if any missing responses are present. When impute = “INN” and random = “TRUE”, the scoring tool will use Item Nearest Neighbor with random (approach 4), the recommended approach according to the results of the present research, to impute missing responses.

The file “MCQ.example.R” provides a few examples of how to organize the dataset and use the wrapper scoring function. All users are encouraged to refer to this file when using our R scoring tool.
